# Acute and chronic eosinophilic pneumonia: an overview

**DOI:** 10.3389/fmed.2024.1355247

**Published:** 2024-04-22

**Authors:** Roberto G. Carbone, Francesco Puppo, Eduardo Mattar, Anja C. Roden, Nikhil Hirani

**Affiliations:** ^1^Department of Internal Medicine, University of Genoa, Genoa, Italy; ^2^Cardiothoracic Imaging, University of Washington, Seattle, WA, United States; ^3^Department of Laboratory Medicine and Pathology, Mayo Clinic, Rochester, MN, United States; ^4^Center for Inflammation Research, The Queen's Medical Research Institute, University of Edinburgh, Edinburgh, United Kingdom

**Keywords:** acute eosinophilic pneumonia, chronic eosinophilic pneumonia, high resolution computed tomography, lung pathology, eosinophilic pneumonias

## Abstract

Acute and chronic eosinophilic pneumonia (AEP and CEP) include a group of rare interstitial lung diseases characterized by peripheral blood eosinophilia, increased eosinophils in bronchoalveolar lavage fluid, or eosinophilic infiltration of lung parenchyma. AEP is characterized by rapid onset, fast response to steroid treatment, and no relapse. CEP is characterized by marked tissue and peripheral blood eosinophilia, rapid response to steroid therapy, and tendency to disease recurrence. In addition, we briefly describe other eosinophilic lung diseases that must be considered in differential diagnosis of AEP and CEP. Eosinophilic pneumonias may be idiopathic or due to known causes such as medications or environmental exposure. At variance with previous reviews on this topic, a particular look in this overview was directed at pathological findings and radiological patterns.

## Introduction

Eosinophilic pneumonias are a heterogeneous group of interstitial lung disease (ILD) characterized by peripheral blood eosinophilia (defined as an eosinophilic count >500 × 10^9^ cells/L), increased eosinophils in bronchoalveolar lavage (BAL) fluid (defined by >5% of eosinophils in the differential cell count), or eosinophilic infiltration of lung parenchyma demonstrated on lung biopsy (1). Eosinophilic pneumonias may be either acute or chronic and may be idiopathic or due to known causes such as medications or environmental exposure. In contrast to Löffler syndrome, a transient lung eosinophilia most frequently associated with parasitic infection, eosinophilic pneumonias are typically more severe and do not resolve without treatment. Here, we report an overview of acute eosinophilic pneumonia (AEP) and chronic eosinophilic pneumonia (CEP). In addition, we briefly describe other eosinophilic lung diseases that must be considered in differential diagnosis of AEP and CEP. Previous reviews have not exhaustively delved into a comprehensive overview of histological images that can better clarify these rare diseases for clinicians. Different morphologic features of pulmonary eosinophilic infiltrates are reported in Table 1. Therefore, we highlighted histological and radiological pictures with related images. High resolution computed tomography (HRCT) is the gold standard imaging procedure for eosinophilic pneumonias. However, a relative overlap of HRCT patterns is observed at least among some types of eosinophilic lung diseases.

**Table 1:** Different morphologic features of pulmonary eosinophilic infiltrates.

Morphologic features	Ancillary tests	Etiology of eosinophilic infiltrate
Eosinophilic infiltrates, possibly associated with organizing pneumonia or diffuse alveolar damage, in a background of smoking-related changes such as intra-alveolar clusters of smoker’s macrophages, emphysema, and smoking-related interstitial fibrosis (Figure 2).		Acute eosinophilic pneumonia (AEP)
Abundant eosinophils and macrophages within airspaces. Interstitial lymphoplasmacytic inflammation is variable. A fibrinous intra-alveolar exudate may be present creating fibroblastic plugs that resemble OP, except for the eosinophilic infiltrate (Figure 4).		Chronic eosinophilic pneumonia (CEP)
Marked infiltrates of eosinophils in bronchial wall and intraluminal mucin with large numbers of eosinophils and possibly Charcot-Leyden crystals (Figure 8).	Grocott methenamine silver (GMS) stain possibly highlights remnants of aspergillus hyphae	Allergic bronchopulmonary aspergillosis (ABPA)
Eosinophilic pneumonia, extravascular granulomatous inflammation including eosinophilic abscesses, eosinophilic vasculitis of arteries, veins, and/or capillaries, possibly with necrotizing vasculitis and/or eosinophilic large airway inflammation (Figure 7).	Serology positive for p-ANCA*, possibly c-ANCA	Eosinophilic granulomatosis with polyangiitis (EGPA)
Intra-alveolar fibroblastic plugs on and around bronchioles with mild lymphoplasmacytic interstitial infiltrate with few eosinophils (Figure 14).		Organizing pneumonia (OP)
Bronchiolocentric infiltrates of epithelioid cells with large nuclei that have grooves, folds, and wrinkles and irregular nuclear border and conspicuous and inconspicuous nucleoli together with increased eosinophils, mixed chronic inflammatory cells and possibly neutrophils. These findings can be seen in association with cystic changes (Figure 6).	CD1a and/or langerin highlight Langerhans cells forming clusters	Pulmonary Langerhans cell histiocytosis (PLCH)
Eosinophilic abscess with parasites.	Parasite serology	Parasites
Eosinophilic pneumonia.	Grocott methenamine silver (GMS) stain	Fungal infection
Macrophages filling alveoli usually in association with an interstitial chronic inflammation and/or fibrosis. Eosinophilic infiltrates may occur.		Desquamative interstitial pneumonia (DIP)

^*^ANCA, Anti-neutrophil cytoplasmic antibodies.

## Acute eosinophilic pneumonia

Acute eosinophilic pneumonia (AEP), also reported as idiopathic acute eosinophilic pneumonia, is a disease of unknown cause. About 400 cases have been reported in the literature and the exact prevalence is unknown. AEP is most common in men between 20 and 40 years of age, smokers, and without a prior history of atopy (2).

Etiology of AEP is poorly understood. It has been hypothesized that it may be determined by an acute hypersensitivity reaction to an inhaled antigen. AEP can be triggered by a variety of respiratory exposures including a recent initiation of tobacco smoking, a change in smoking habits, reintroduction to smoking or even short-term passive smoking, vaping, and environmental exposure to inhaled contaminants such as dust from the World Trade Center collapse or among military personnel in Iraq. AEP onset may also be associated with several medications like antibiotics, nonsteroidal anti-inflammatory drugs, and selective serotonin-reuptake inhibitors as well as with parasitic, fungal, and viral infections (3–6).

Pathogenesis of AEP is not completely understood. A proposed model of pathogenesis suggests that airway and epithelial damage may induce interleukin (IL)-33 production leading to the recruitment of eosinophils, infiltration, and degranulation of these cells and subsequently to an inflammatory process responsible for clinical symptoms (1).

Idiopathic AEP clinically mimics severe community acquired pneumonia and acute respiratory distress syndrome (ARDS), with dyspnea, fever, nonproductive cough, hypoxemia, and pulmonary infiltrates occurring within 7 days (2). Severe hypoxemic respiratory failure requiring mechanical ventilation is common (2). AEP should be considered in patients presenting with an ARDS-like syndrome without a clear precipitating cause (2, 7).

### Diagnostic criteria

Acute eosinophilic pneumonia is characterized by acute onset with fever (≤1 month), bilateral diffuse infiltrates, PaO_2_ ≤ 60 mmHg or oxygen saturation ≤ 90% on room air, BAL eosinophilia (≥ 25% eosinophils) or eosinophilic pneumonia at lung biopsy, and absence of other known causes of eosinophilic pneumonia (8).

### Imaging

**Figure 1:**
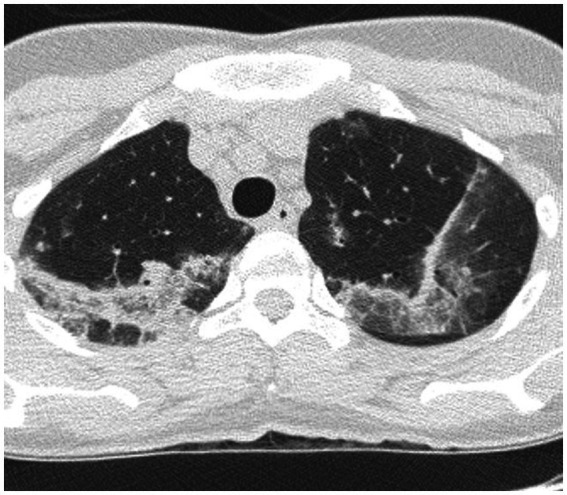
HRCT findings in acute eosinophilic pneumonia. Bilateral patchy areas of ground-glass opacity in both lungs and interlobular septal thickening.

Imaging typically reveals symmetric alveolar and interstitial infiltrates resembling ARDS; pleural effusions may occur in up to 70% of patients (9). High resolution computed tomography (HRCT) features of AEP include: (1) ground glass opacities; (2) interlobular septal thickening; (3) pleural effusion (60–100% of cases); (4) thickening of bronchovascular bundles; (5) air space consolidations; and (6) centro-lobular nodules (10) (Figure 1). Notably, AEP imaging may mimic cardiogenic pulmonary edema or ARDS.

### Laboratory findings

Leukocytosis is common but blood eosinophilia is often absent at disease onset and may develop later in the course of the disease (2). BAL containing at least 25% eosinophils in the appropriate clinical setting is typically diagnostic, though this percentage may be lower in patients who have received corticosteroids prior to bronchoscopy (11). BAL IL-5 levels have been reported to be higher in AEP than in chronic eosinophilic pneumonia, despite the lower level of eosinophilia (12).

### Lung function tests

Pulmonary function tests may reveal restriction and a reduced diffusing capacity for carbon monoxide (DLCO), which resolves after therapy (7).

### Pathology

**Figure 2:**
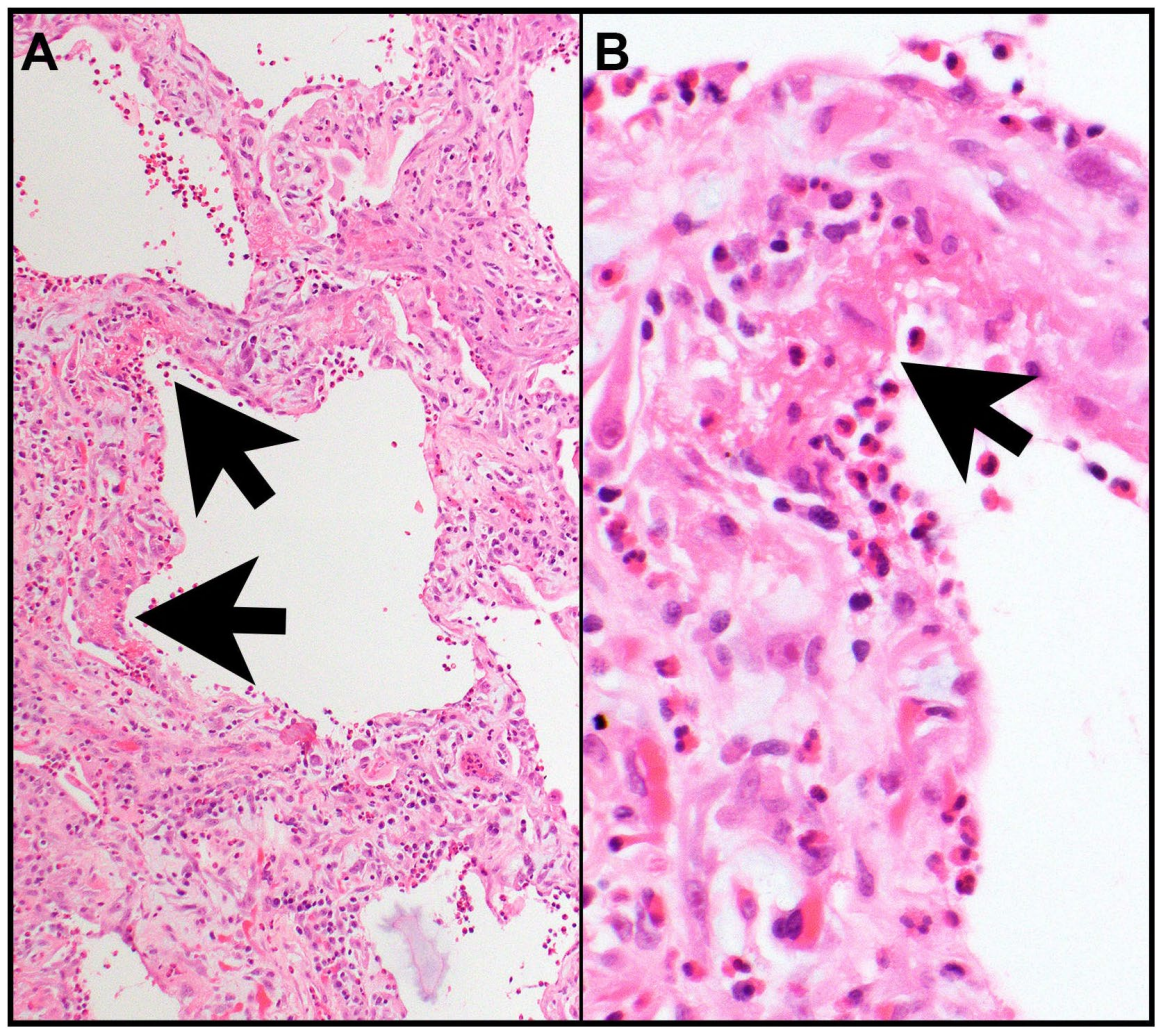
Acute eosinophilic pneumonia. **(A)** The interalveolar septa are thickened by proliferating fibroblasts (features of organizing diffuse alveolar damage) with many intermixed eosinophils and type II pneumocyte hyperplasia and are focally lined by hyaline membranes (arrows) (features of acute diffuse alveolar damage). **(B)** Numerous eosinophils are in the alveolar space and within hyaline membrane (arrow) and interalveolar septa. Magnification: H&E × 10 **(A)**, ×400 **(B)**.

A biopsy is not necessary for diagnosis but, when performed, reveals interstitial edema and eosinophilic infiltration of bronchial walls, interstitium, and/or alveolar spaces. The presence of diffuse alveolar damage (DAD) with hyaline membranes and/or interstitial fibroblasts and eosinophilic infiltrates suggests AEP (Figures 2A,B) (1, 13).

### Treatment

Patients typically respond dramatically to corticosteroids with clinical improvement usually seen within 48 h and resolution of infiltrates within 1 month. While the optimal corticosteroid regimen for AEP has not been determined, high intravenous doses (up to 500 mg/day methylprednisolone) are required for severe respiratory failure that may be switched to oral prednisone (40–60 mg/day) when improvement occurs. Oral prednisone is typically continued for 2–4 weeks followed by tapering over the next several weeks (2, 14). Recurrence of AEP after treatment is uncommon.

## Chronic eosinophilic pneumonia

Chronic eosinophilic pneumonia (CEP) was first described by Liebow and Carrington (15) and has been estimated to account for 1–3% of interstitial lung diseases. CEP is characterized by marked tissue and peripheral blood eosinophilia (8, 16) and its diagnosis is based on clinical symptoms and laboratory findings after exclusion of other eosinophilic lung diseases (17).

Chronic eosinophilic pneumonia is more frequent in women than in men with a 2:1 predominance and most commonly occurs between 30 and 45 years of age (18). Patients with CEP are typically nonsmokers, up to two-thirds have a history of adult-onset asthma and about half had prior atopy or allergic rhinitis (18).

Chronic eosinophilic pneumonia usually produces subtle and progressive respiratory symptoms over weeks to months whereas frank respiratory failure is rare. Dyspnea, cough, and constitutional symptoms including low-grade fever, night sweats, malaise, and unintentional weight loss are common. Respiratory symptoms are usually present for more than 2 weeks and extra-pulmonary symptoms are uncommon (18).

Etiology and pathogenesis of CEP are not completely known. Lung infiltration by eosinophils expressing activation markers and release of proinflammatory cytokines by infiltrating eosinophils have been proposed as possible pathogenetic mechanisms leading to CEP development. Recently, a possible pathogenetic role for clonal blood and lung T cell expansion has been also suggested (8).

**Figure 3:**
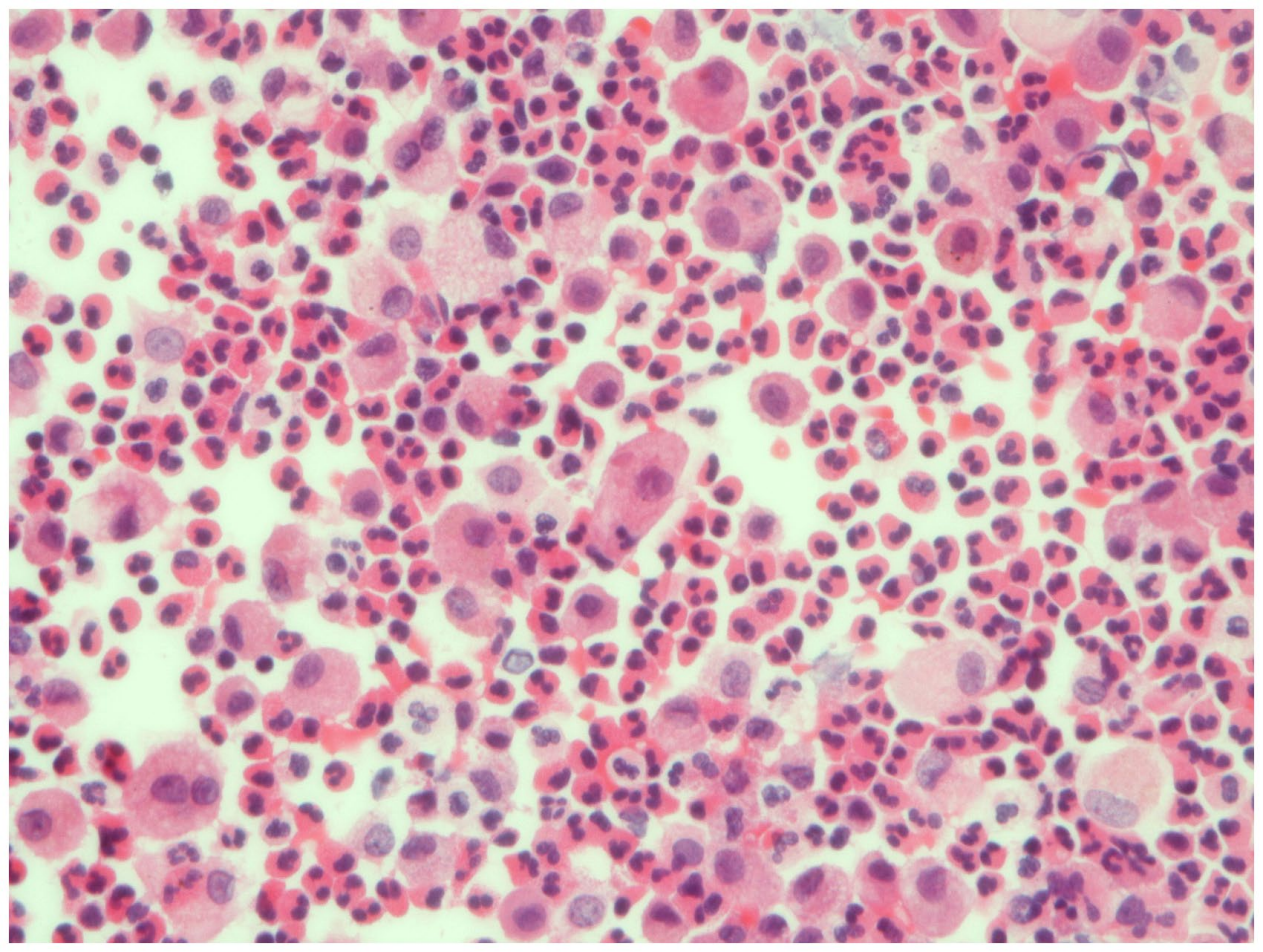
Bronchoalveolar lavage. Detection of numerous eosinophils in BAL, distinguished bright pink granular cytoplasm, consistent with a diagnosis of eosinophilic pneumonia.

The combination of peripheral consolidation at imaging, blood eosinophilia, and BAL eosinophilia (Figure 3), and response to steroid treatment are often sufficient for the diagnosis, avoiding the need to perform lung biopsy. However, it is sometimes difficult to differentiate CEP from organizing pneumonia (OP) because eosinophils could be slightly increased in BAL of cryptogenic OP and HRCT features may be similar. However, in CEP, consolidations are less frequently peribronchovascular and less frequently show a reverse halo sign, as compared to OP (19–22).

### Diagnostic criteria

Chronic eosinophilic pneumonia is characterized by respiratory symptoms present for 2–4 weeks, diffuse pulmonary alveolar consolidation with air bronchogram and/or ground-glass opacities, BAL eosinophilia (≥ 40% eosinophils) or peripheral blood eosinophilia (≥1,000/mm^3^), and absence of other known causes of eosinophilic pneumonia (8).

### Imaging

**Figure 4:**
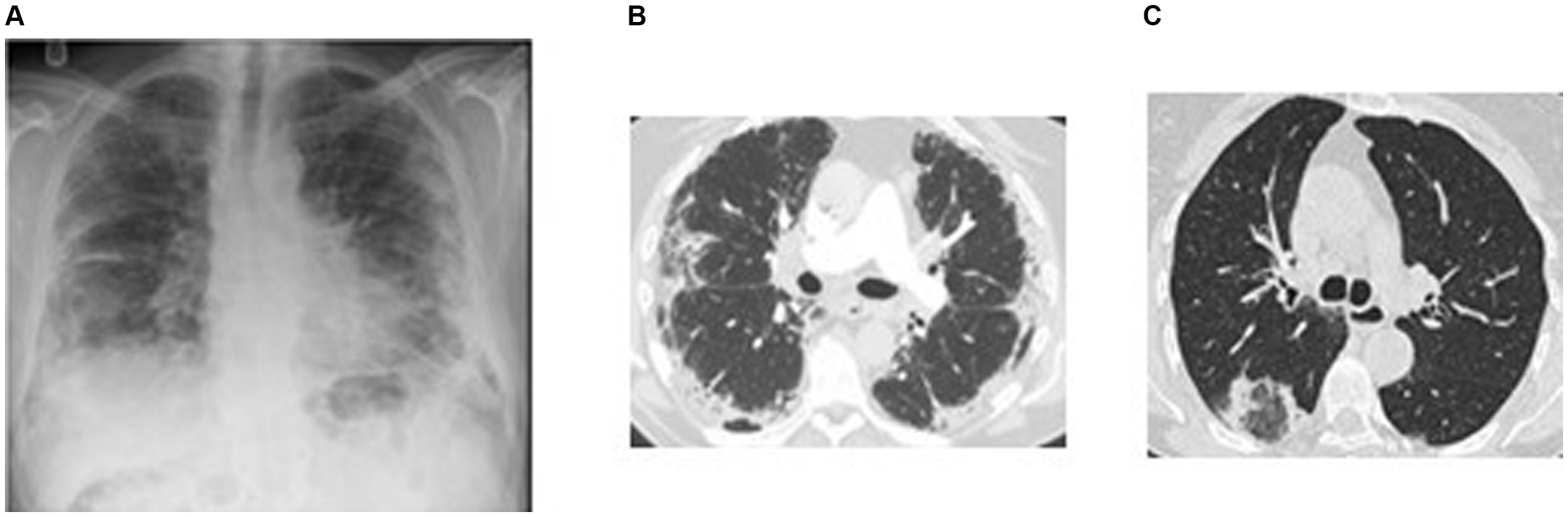
Imaging in chronic eosinophilic pneumonia. **(A)** Plain chest radiograph in a subject with chronic eosinophilic pneumonia showing peripheral air-space opacification in the mid and lower zones. **(B)** Chest HRCT scan of the same subject showing peripheral sub-pleural consolidation and ground glass changes with sparing of the central areas. This radiographic feature is typical for eosinophilic pneumonia. **(C)** Chest HRCT scan in a different subject showing consolidation encircling an area of ground glass opacification resembling a reversed halo (attol sign). This feature is seen in chronic eosinophilic pneumonia but also in a number of other pathologies including COP, sarcoidosis, vasculitis, and infection.

Chest imaging is often helpful, though the “characteristic” finding of dense peripheral infiltrates on plain chest *x*-ray, which have been described as a “photographic negative” of pulmonary edema, occur in less than 50% of cases (20, 23). HRCT findings in CEP typically reveal dense and patchy areas of consolidation and ground glass affecting the outer two-thirds of the mid to upper lung fields bilaterally (Figures 4A,B). Less common radiographic manifestations include reverse halo (atoll) sign (Figure 4C), septal thickening, nodular infiltrates, mediastinal adenopathy, bronchial wall thickening, and pleural effusion (24). When symptoms have been present for months, even after treatment, imaging may show linear band-like opacities parallel to pleural surface or lobar atelectasis. The linear bands are not interrupted by the pulmonary fissures.

### Laboratory findings

Blood eosinophilia occurs in 66–95% of patients, with eosinophils representing up to 20–30% of the leukocyte differential count. Erythrocyte sedimentation rate and C-reactive protein are typically elevated. Serum IgE levels may be elevated in about half of the patients. BAL eosinophilia, usually above 40% of total cells pre-corticosteroid treatment, is always present and in the appropriate clinical setting is often diagnostic. IL-5 levels are elevated in both serum and BAL, but do not necessarily correlate with the degree of eosinophilia in lungs (25).

### Lung function tests

Pulmonary function testing may reveal obstruction, restriction, or normal physiology. Approximately half of CEP patients have airflow obstruction and the remaining have a restrictive ventilatory defect. DLCO is reduced in most patients.

### Pathology

**Figure 5:**
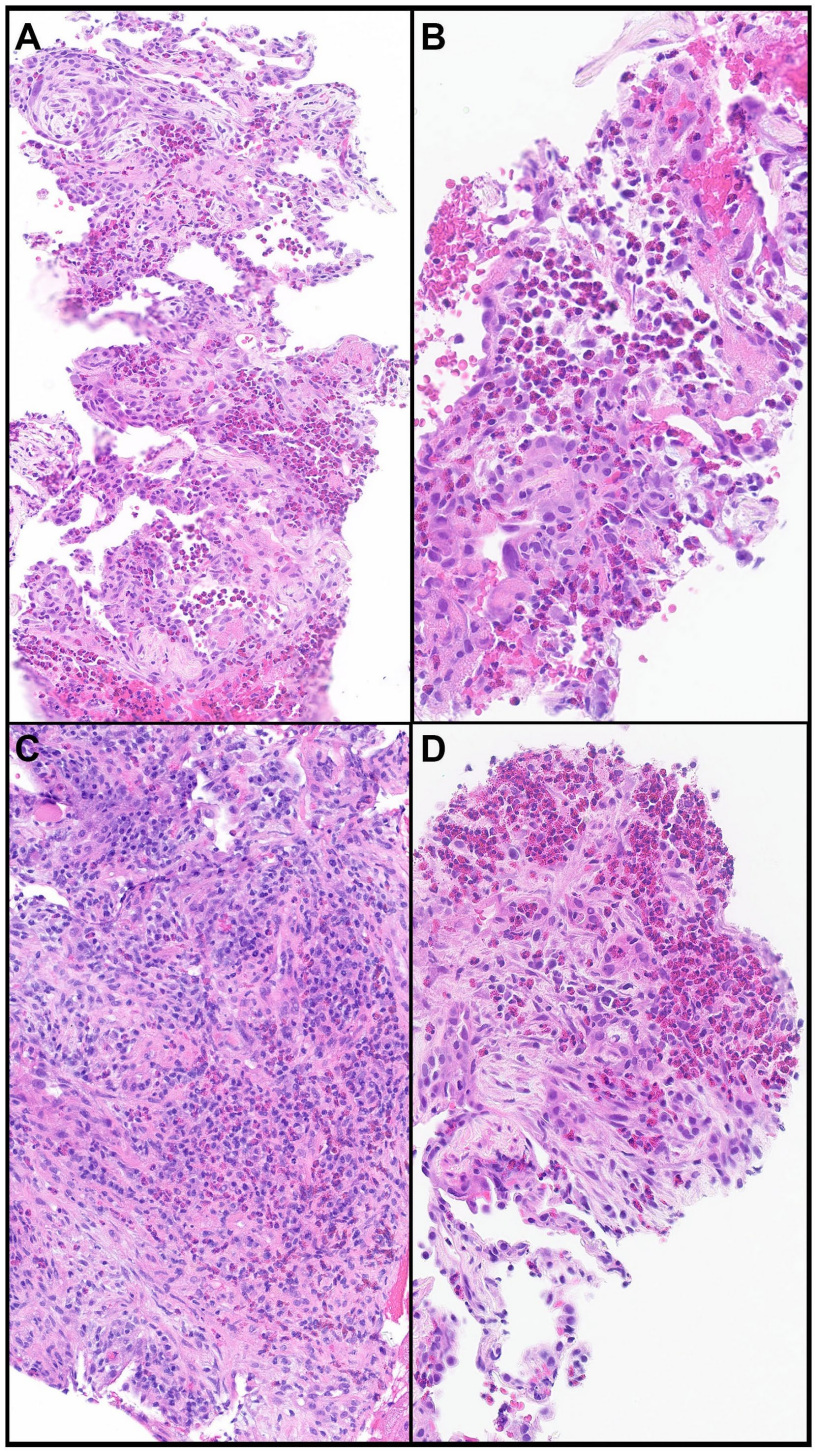
Chronic eosinophilic pneumonia. **(A)** Numerous eosinophils scattered and in clusters on a background of organizing pneumonia and thickened interalveolar septa. **(B)** Eosinophils filling an alveolar space. **(C)** Eosinophils together with other chronic inflammatory cells in a background of organizing pneumonia. **(D)** Clusters of eosinophils in a background of organizing pneumonia. Magnification: H&E × 10 **(A)**, ×40 **(B,D)**, and ×20 **(C)**.

Lung biopsy is not required when HRCT pattern, laboratory data, and response to steroid treatment support CEP diagnosis. The histopathological findings of CEP include large numbers of eosinophils infiltrating alveolar spaces and interalveolar septa (Figures 5A–D). These infiltrates can be accompanied by other chronic inflammatory cells such as macrophages and lymphocytes (Figure 5C). Large clusters of eosinophils possibly associated with nuclear debris can form eosinophilic micro-abscesses. Eosinophilic granules and possibly Charcot-Leyden crystals may occur, specifically in allergic bronchopulmonary aspergillosis. The architecture of the background lung parenchyma is preserved (13). Eosinophilic abscesses, airway involvement with non-necrotizing perivascular inflammation, type II pneumocyte hyperplasia, interstitial lymphocytes, and organizing intra-alveolar fibrin may also be present. Foci of organizing pneumonia (OP) are frequently noted (Figures 5A,C,D). Rarely, eosinophilic pneumonia may present with DAD or acute fibrinous OP, usually in the acute or subacute setting (26).

**Figure 6:**
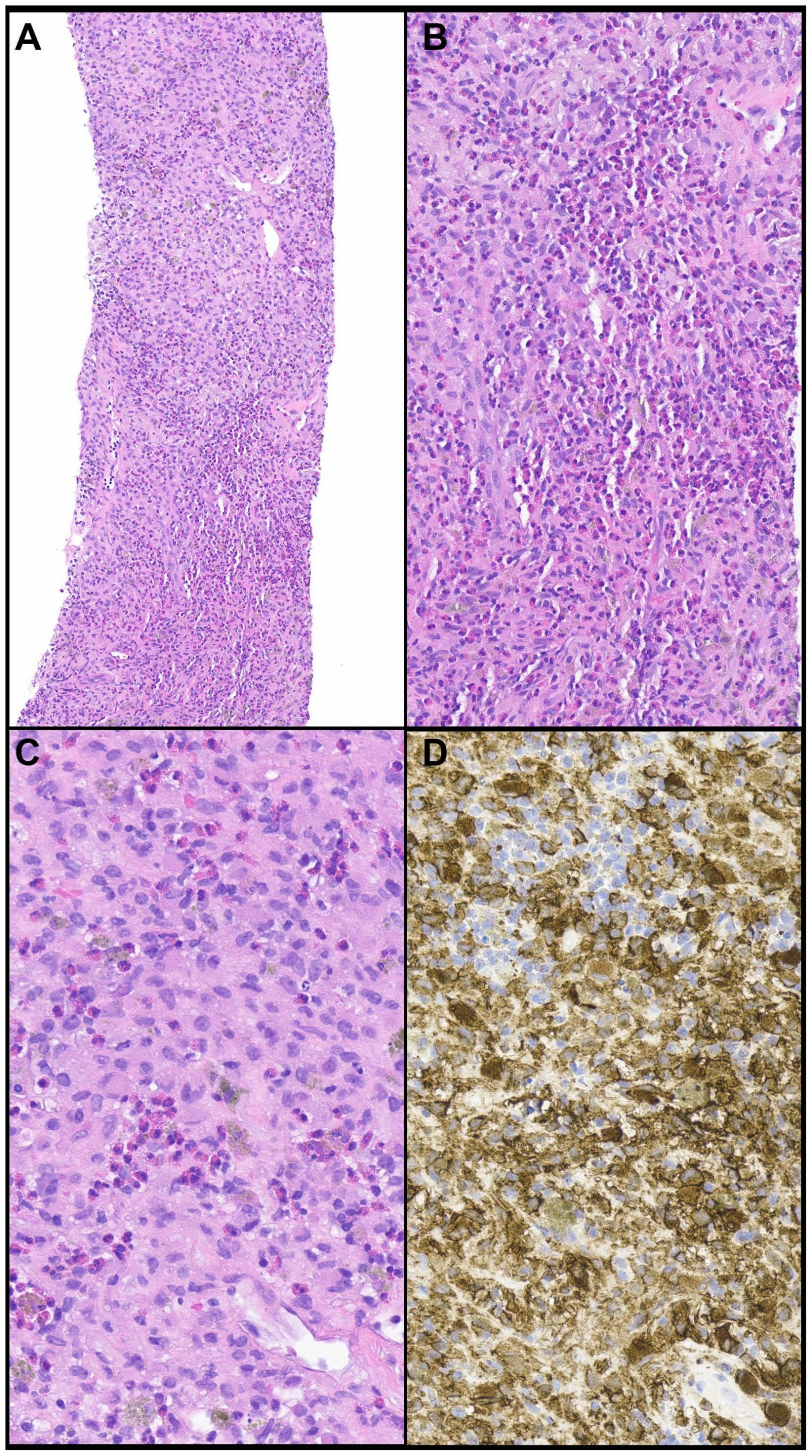
Pulmonary langerhans cell histiocytosis. **(A,B)** A cellular infiltrate is comprised of numerous eosinophils and large cells. **(C)** The large cells (Langerhans cells) have round to oval nuclei that contain grooves or folds or are wrinkled with conspicuous or inconspicuous nucleoli. **(D)** The large cells express CD1a, a hallmark feature of PHLC. Magnification: H&E ×10 **(A)**, ×400 **(B–D)**.

**Figure 7:**
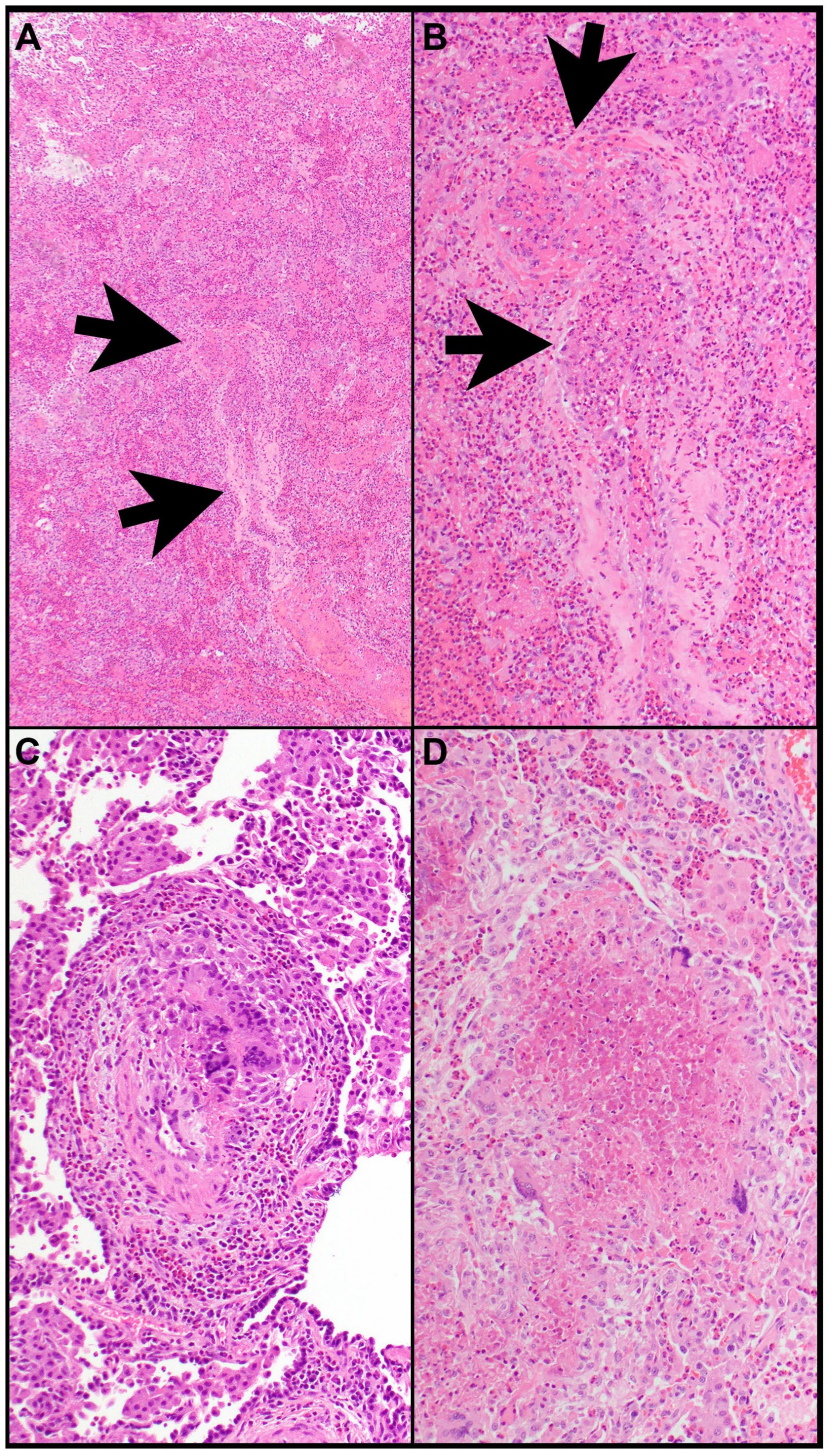
Eosinophilic granulomatosis with polyangiitis. **(A)** Airspaces are filled with eosinophils. The wall of a vessel (arrows) is infiltrated by eosinophils **(B)**. **(B)** That vessel shows focal fibrinous necrosis with numerous eosinophils (arrows). Note that eosinophils are also filling airspaces adjacent to the vessel. **(C)** The wall of a vessel contains numerous eosinophils and clusters of multinucleated giant cells. **(D)** A necrotizing granuloma is filled with eosinophilic necrosis that contains scattered eosinophils and is rimmed by epithelioid histiocytes and multinucleated giant cells. Magnification: H&E ×4 **(A)**, ×40 **(B–D)**.

**Figure 8:**
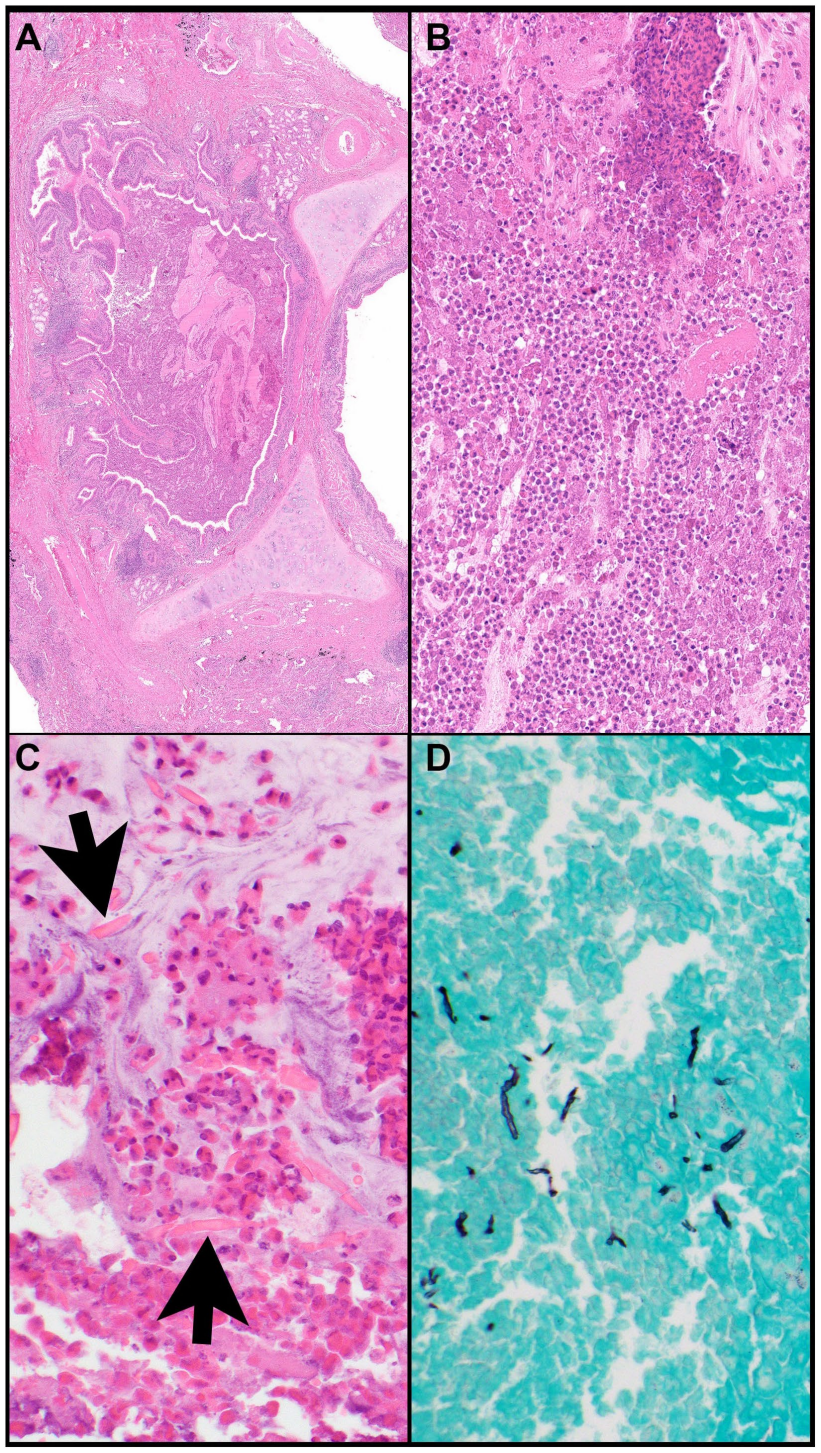
Allergic bronchopulmonary aspergillosis. **(A)** A bronchus (note, cartilage) is filled with eosinophilic material and mucin and is surrounded by inflammatory infiltrates. **(B)** The bronchus is filled with eosinophils, eosinophilic debris, proteinaceous fluid, and mucin. **(C)** Charcot-Leyden crystals (arrows) are together with clusters of eosinophils within mucin (“allergic mucin”). **(D)** A Grocott methenamine silver (GMS) stain highlights remnants of hyphae. Magnification: H&E ×4 **(A)**, ×40 **(B,C)**, and GMS ×40 **(D)**.

In most biopsies that show morphologic features of eosinophilic pneumonia, an etiology cannot be established and requires clinical correlation. However, certain morphologic features reveal potential causes or differential diagnoses of eosinophilic pneumonia including Pulmonary (or pulmonary involvement of systemic) Langerhans cell histiocytosis (PLCH) (Figures 6A–D), desquamative interstitial pneumonia, eosinophilic granulomatosis with polyangiitis (Figures 7A–D), parasitic infection, or allergic bronchopulmonary aspergillosis (Figures 8A–D). Importantly, treatment with steroids before tissue biopsy may mask eosinophilic pneumonia, as eosinophils will vanish relatively quickly following steroid treatment.

### Treatment

Prompt steroid treatment improves CEP prognosis (20). In contrast to AEP however, long-term low dose oral corticosteroid therapy is required to prevent relapse and to obtain full remission. Corticosteroids are the therapy of choice for CEP, and patients typically respond dramatically (27). While the optimal corticosteroid regimen for CEP has not been determined, treatment usually starts with prednisone at a dose of 0.5 mg/kg/day for about 4–6 weeks, at which time radiographic abnormalities generally resolve. The dose may then be tapered to 0.25 mg/kg/day, which is continued for another 8 weeks, followed by further tapering by 5 mg every month (27). Relapse is common and up to 50% of patients may require long-term low dose oral corticosteroids or high-dose inhaled corticosteroids to maintain disease control. Relapses are often treated with high doses of corticosteroids (27). Recently, biological agents, such as the anti-IgE antibody (omalizumab), the anti-IL-5 antibody (mepolizumab), and the anti-IL-5 receptor antibody (benralizumab) could be alternative CEP steroid-sparing treatments (17).

Distinctive characteristics of AEP and CEP and cohort studies of AEP and CEP patients are reported in Tables 2, 3, respectively.

**Table 2:** Distinctive characteristics of acute eosinophilic pneumonia (AEP) and chronic eosinophilic pneumonia (CEP).

	Epidemiology	Clinical characteristics					Chest imaging	Treatment	Relapse
		Onset	History of asthma	Smoking history	Blood eosinophilia	BAL eosinophilia			
AEP	~ 400^*^	< 1 month	No	> 75%	No	>25%	Bilateral areas of ground-glass, consolidation, interlobular septal thickening, and pleural effusion	Corticosteroids (2–4 weeks)	No
CEP	1–3%^**^	>2–4 weeks	Yes	10%	Yes	>25%	Homogeneous peripheral consolidation	Corticosteroids (long term) anti-IgE and anti-IL5 mAb	Yes

^*^Reported cases; ^**^Interstitial lung disease; BAL, Bronchoalveolar lavage; mAb, monoclonal antibody.

**Table 3:** Cohort studies of acute eosinophilic pneumonia (AEP) and chronic eosinophilic pneumonia (CEP).

	Author	No of cases	Age—year (mean)	Sex (female)^*^	Smoker^*^	Asthma^*^
AEP						
	Philit	22	29	41	36	No
	Shorr	18	22	11	100	No
	Uchiyama	33	19	30	97	9
	Rhee	137	20	0	98	2
	Jhun	85	21	0	99	N/A
	Sine	43	25	7	91	N/A
	De Giacomi	36	47	56	99	N/A
CEP						
	Suzuki	133	58	63	3	50
	Ishiguro	73	56	64	34	48
	Oyama	44	61	67	2	61
	Marchand	62	45	68	6	52
	Marchand	53	43	64	13	64
	Durieu	19	51	84	0	37
	Naughton	12	38	100	8	33

See Ref. 1, 17, ^*^Data are expressed as percentage.

## Other eosinophilic lung disease

Eosinophilic pneumonias are often mistaken for the more common conditions respiratory infection, acute respiratory distress syndrome, or cryptogenic organizing pneumonia (COP) due to shared symptomatology and radiographic findings. However, these conditions do not have pronounced or persistent eosinophilia. The diagnosis of idiopathic types of eosinophilic pneumonia needs to exclude those of known causes. Eosinophilic syndromes that form the differential diagnosis of AEP and CEP are listed in Table 4.

**Table 4:** Other eosinophilic lung disease.

*Eosinophilic lung diseases of known causes*
^*^Löffler syndrome (Simple pulmonary eosinophilia)
^*^Allergic Broncho-Pulmonary Aspergillosis (ABPA)
^*^Drug induced Eosinophilic Pneumonia
*Eosinophlic lung diseases with extrapulmonary involvement*
^*^Hypereosinophilic syndrome
^*^Eosinophilic Granulomatosis with Polyangiitis (EGPA)—Churg Strauss Syndrome
*Interstitial lung diseases that may show lung eosinophilia*
^†^Acute respiratory distress syndrome (ARDS)
^†^Cryptogenic Organizing Pneumonia (COP)
^‡^Pulmonary Langerhans Cell Histiocytosis (PLCH)
^‡^Idiopathic Pulmonary Fibrosis
^‡^Sarcoidosis

^*^Presence of lung and circulating eosinophilia. †Presence of lung but not circulating eosinophilia. May have similar clinical and radiological features to eosinophilic pneumonia. ^‡^Presence of lung but not circulating eosinophilia but readily distinguishable from eosinophilic pneumonia by clinical and radiological features.

### Eosinophilic lung diseases of known causes

#### Lӧffler syndrome

**Figure 9:**
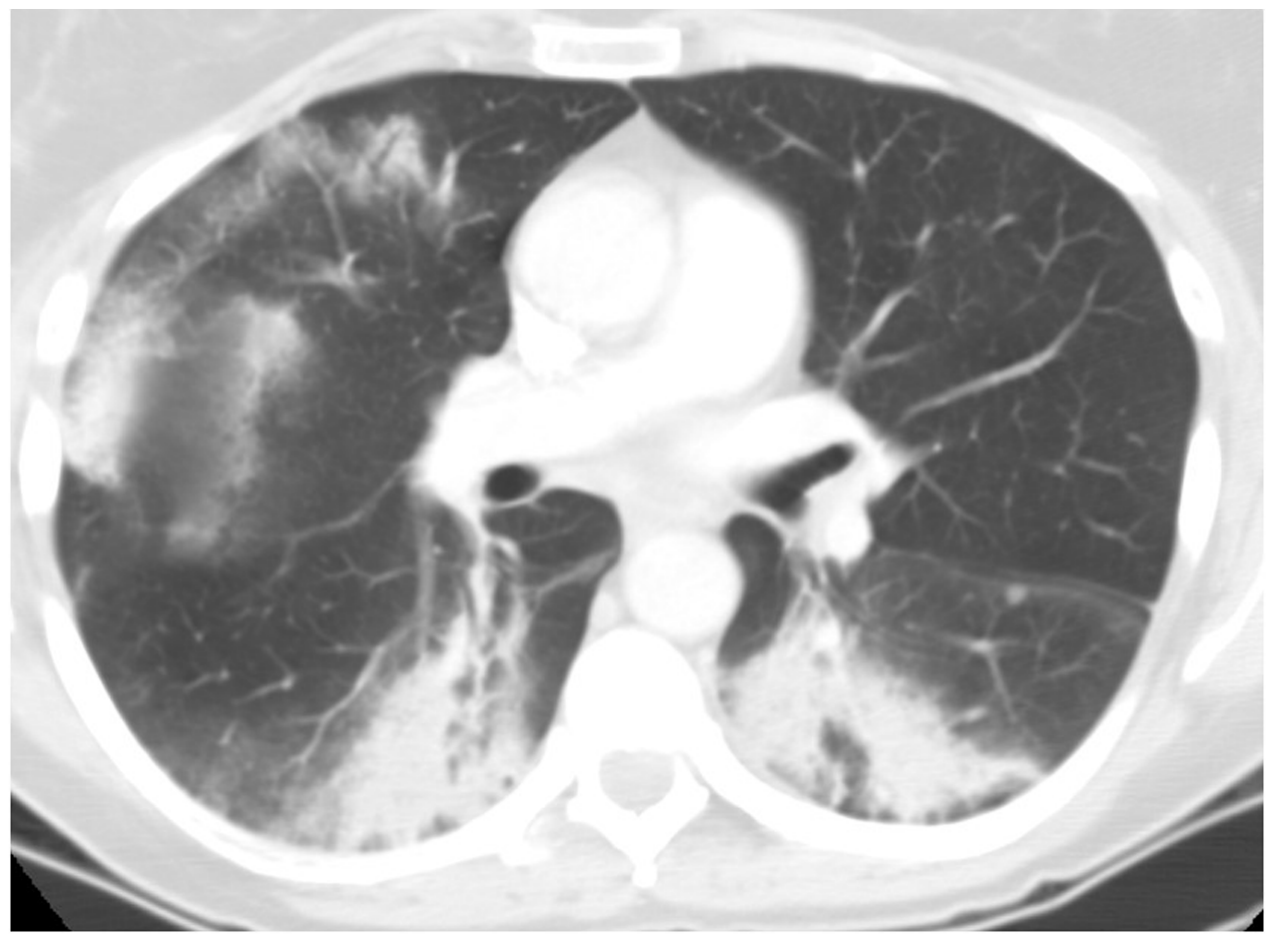
HRCT findings in Loeffler syndrome. Marked fleeting infiltrates associated with bilateral sites of consolidation, ground glass opacities with peri-lobular and peripheral lung distribution.

Lӧffler syndrome, also known as simple pulmonary eosinophilia (SPE), is an eosinophilic pulmonary disease characterized by transient pulmonary infiltrates, absent or mild respiratory symptoms, and peripheral blood. Prevalence of SPE in asymptomatic healthy subjects was reported to be about 0.9%. HRCT shows migratory and fleeting, non-segmental air space opacifications, which may be unilateral or bilateral with predominantly peripheral distribution. Solid nodules surrounded by ground-glass attenuation (halo sign) may be present. Pleural effusions and lymphadenopathy are not present (Figure 9). A Löffler-like syndrome may occur after helminthic infections with *Ascaris lumbricoides* and *Anchilostoma duodenale*. Microscopic examination, and serological studies should be undertaken in high-risk individuals, however, the etiologic agent is not identifiable in up to one third of patients. Löffler syndrome often spontaneously recovers within 1 month and corticosteroids treatment is suggested when resolution does not occur (28, 29).

#### Allergic broncho-pulmonary aspergillosis

**Figure 10:**
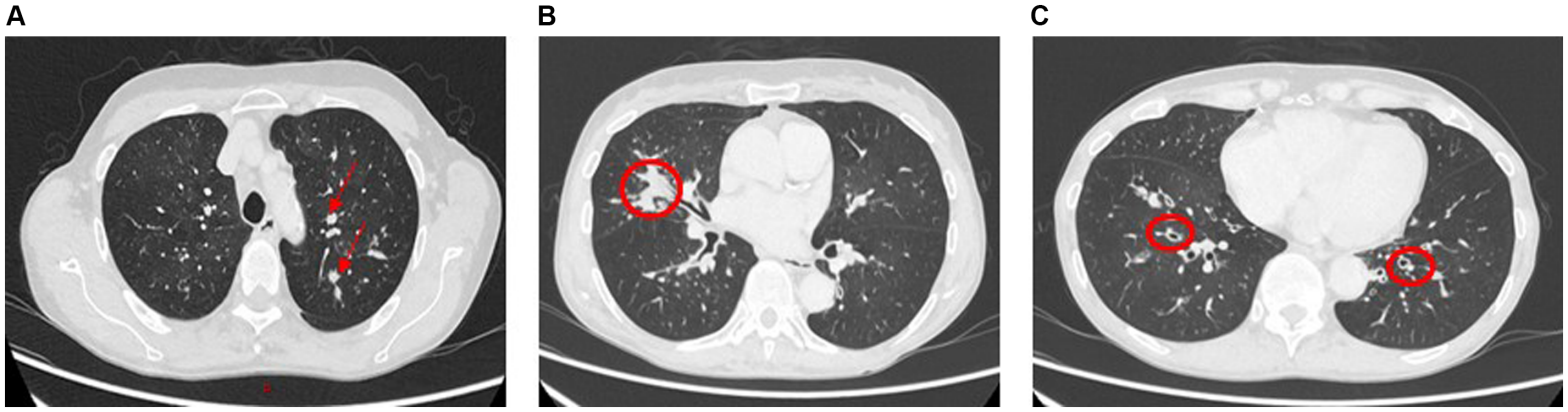
Allergic bronchopulmonary aspergillosis (ABPA). Panels **(A,B)**: Left upper lobe and right middle lobe airway showing mucous plugging (arrows and circled) giving nodular or mass-like appearances. Further impaction of larger airways in ABPA commonly leads to partial or total lobar collapse. Panel **(C)**: Lower lobe relatively proximal bronchiectasis (circled).

Allergic bronchopulmonary aspergillosis (ABPA) is a hypersensitivity reaction to *Aspergillus* species most commonly seen in patients with asthma and cystic fibrosis. HRCT patterns show central bronchiectasis, hyperattenuating mucous plugs, pulmonary nodules, and infiltrates (Figures 10A–C). A combination of peripheral eosinophilia raised total and Aspergillus-specific IgE and typical radiological findings in the right clinical setting is diagnostic for ABPA.

Lung biopsy is not required for diagnosis, but the classic morphologic features include marked infiltrates of eosinophils in the wall of large airways together with intraluminal mucin containing sheets and clusters of eosinophils and possibly Charcot-Leyden crystals (“allergic mucin”) (Figures 8A–C). A fungal stain such as Grocott Methenamine silver (GMS) stain may or may not reveal remnants of fungal hyphae. The presence of fungal hyphae is not required as ABPA is a hypersensitivity reaction to the fungus implicating exposure to the fungus but not necessarily infection.

Treatment of ABPA is conventionally inhaled and oral corticosteroids, but there is a role for antifungal treatment and monoclonal antibodies directed for example to IgE and IL5 in selected cases.

#### Drug induced eosinophilic pneumonia

**Figure 11:**
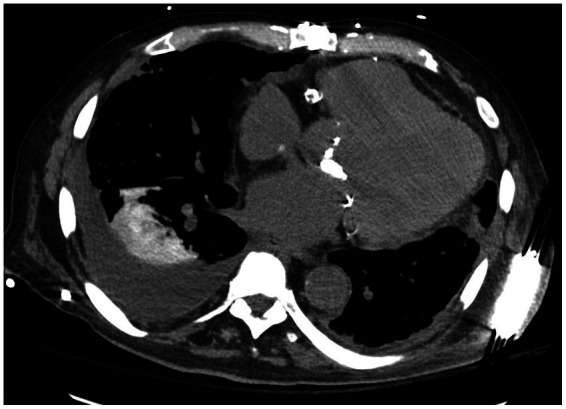
HRCT findings in amiodarone pulmonary toxicity. Lower lobe bilateral basilar infiltrates and pleural effusions especially on the right lung.

Drug induced eosinophilic pneumonia represents an important subset of patients who present with eosinophilia and eosinophilic pulmonary infiltrates causing severe respiratory syndrome (Figure 11). Numerous drugs are associated with eosinophilic pneumonia including antibiotics and anti-inflammatory non-steroidal drugs or cardiac anti-arhythmic drugs such as amiodarone. Radiotherapy is also implicated in eosinophilic pneumonia (Table 5) (30–35).

**Table 5:** A selection of drugs inducing eosinophilic pneumonia.

Drug^*^	Category
Amiodarone	Anti-arrhythmic
Amphotericin B	Anti-mycotic
Ethambutol	Anti-mycobacterial
Isoniazid	Anti-mycobacterial
Diphenylhydantoin	Neurologic
Carbamazepine	Neurologic
Trazodone	Neurologic
GM-CSF^#^	Immunomodulating
Bleomicin	Chemotherapic
Methotrexate	Chemotherapic
Procarbazine	Chemotherapic
Etoposide	Chemotherapic
Minocycline	Antibiotic
Tetracycline	Antibiotic
Sulphonamides	Antibiotic
Nitrofurantoin	Antibiotic
Sulfasalazine	Antibiotic
Mitomycin-C	Antibiotic
Para-aminosalicylic acid	Anti-inflammatory
NSAIDs^##^	Anti-inflammatory
Penicillamine	Anti-inflammatory

^#^Granulocyte-macrophage colony-stimulating factor; ^##^Nonsteroidal anti-inflammatory drugs.

The principal tools for the diagnosis of drug-induced eosinophilic pneumonia are like idiopathic AEP and CEP and so a high index of suspicion is required especially if there is a temporal relationship with the drug, although eosinophilic pneumonia can present with chronic drug use. The pathophysiology of drug-induced eosinophilic pneumonia is related to the production of an antigen by alveolar macrophages leading to the recruitment of T–helper 2 lymphocytes and subsequent release of interleukin 2. Of interest, amiodarone-induced CEP is caused from a combination of cytotoxic effect to type-II pneumocytes, immune-mediated mechanisms, and activation of the angiotensin enzyme system (32). A common pathogenetic pathway may underlie AEP and CEP caused by different drugs. Generally, patients affected by drug-induced eosinophilic pneumonia demonstrate a good response to steroid treatment and drug-cessation and few require mechanical ventilation.

### Eosinophlic lung diseases with extrapulmonary involvement

#### Hypereosinophilic syndrome

Hypereosinophilic syndrome is a clonal disease characterized by peripheral blood eosinophilia >15 × 10^9^/L persisting for more than 6 months. Asthma-symptoms and lung involvement are features but uncommon. Notably, there are two clonal variants of hypereosinophilic syndrome namely a myeloproliferative form, which can rarely transform into acute myeloid leukemia or lymphoblastic leukemia, and a lymphoproliferative syndrome. HRCT shows patchy ground-glass attenuation, consolidation, and small nodules.

#### Eosinophilic granulomatosis with polyangiitis—Churg Strauss syndrome

**Figure 12:**
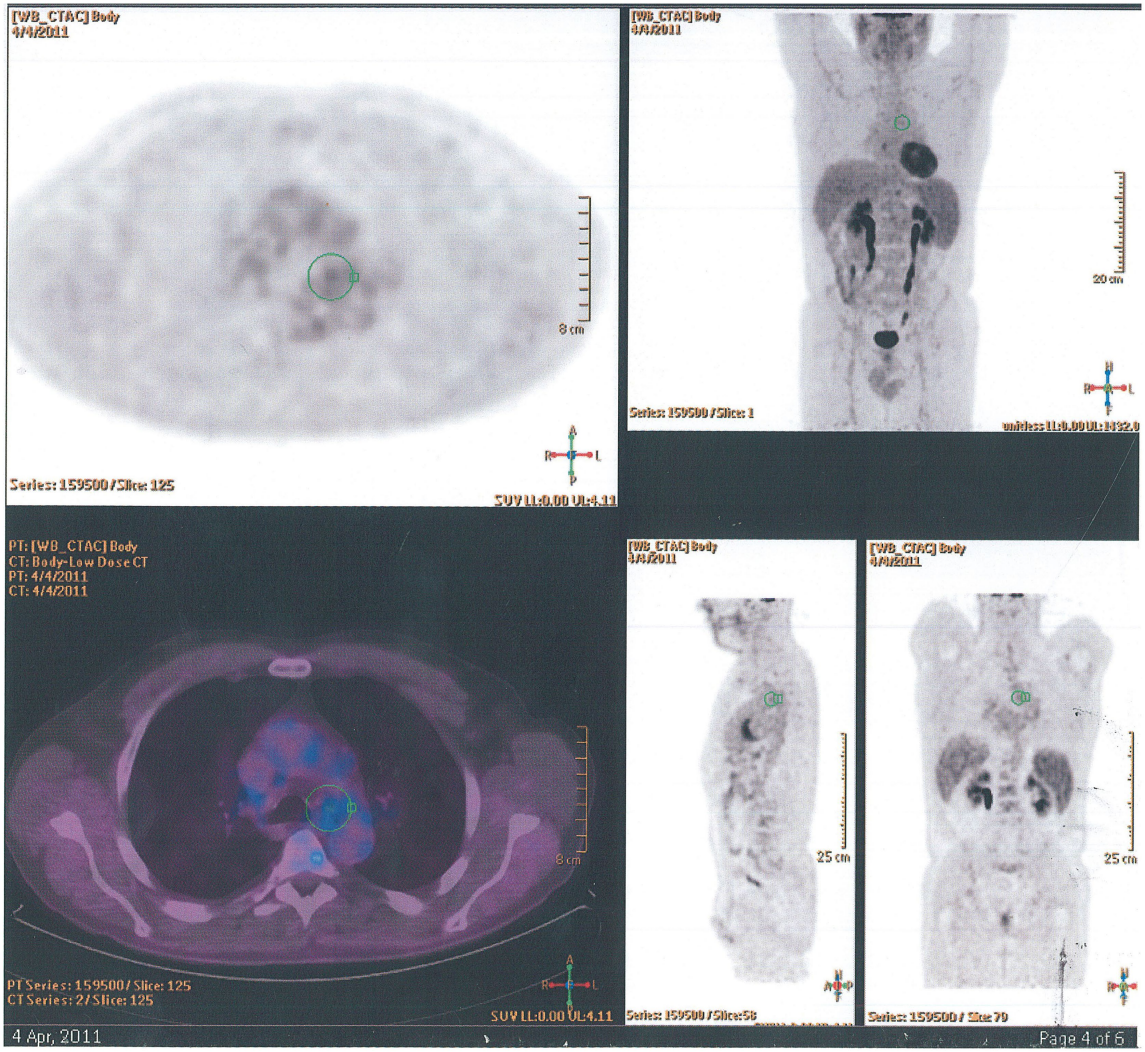
^18^F-FDG PET/CT findings in eosinophilic granulomatosis with polyangiitis. Axial, coronal, and sagittal images show paracardial nodular granulomatous infiltrate.

Eosinophilic granulomatosis with polyangiitis is a small and medium-vessel vasculitis and may present with multiorgan involvement including skin, respiratory tract, and bowel, renal, cardiac, and ocular disease. The characteristic features include asthma, sinusitis, neuropathy, and lung and/or blood eosinophilia. About 50% of patients with EGPA are positive for anti-neutrophil-cytoplasmic-antibodies (ANCA). HRCT often shows diffuse bilateral peripheral ground-glass infiltrates, bronchial wall and septal thickening, and bronchocentric non-cavitating nodular infiltrates. Pleural effusion occurs in less than a third of patients. Positron emission tomography using ^18^F-2-fluoro-2-deoxyglucose/computed tomography (^18^F-FDG PET/CT) shows nodular granulomatous infiltrates (Figure 12). The diagnosis is often made through a combination of clinical and serological features but supported by biopsy evidence from skin, nerve, or kidney. Lung biopsy is infrequently needed, but morphologically EGPA classically shows features of eosinophilic pneumonia as discussed above, necrotizing granulomas with eosinophilic necrosis that may contain scattered intact of apoptotic eosinophils, eosinophilic abscesses, eosinophilic vasculitis of arteries, veins, and/or capillaries, possibly with fibrinous necrosis of the vessel wall and eosinophilic large airway inflammation (Figures 7A–D). Induction treatment of EGPA is with corticosteroids and immunosuppression conventionally with cyclophosphamide or rituximab. The maintenance treatment and prognosis are dependent on defined prognostic factors at presentation (36–38).

### Interstitial lung diseases that may show lung eosinophilia

#### Acute respiratory distress syndrome

Acute respiratory distress syndrome (ARDS) is an inflammatory lung disease caused by fluid buildup in small alveoli occurring in sepsis, aspiration pneumonia, Sars-Cov-2 infection, pancreatitis, and blood transfusion. Major trauma and burns, inhalational injury, and drug overdose, are other possible causes of ARDS.

Acute respiratory distress syndrome develops in few hours to days from the trigger event and can worsen rapidly. Most lung lobes may be affected, and patients frequently need admission to Intensive Care Unit (ICU). Deep vein thrombosis and pneumothorax are the principal ARDS complications. Diagnostic procedures include chest—*x*-ray, echocardiogram, breathing tests, and CT scan.

Acute respiratory distress syndrome in usually treated in ICU using mechanical ventilation in association with oxygen therapy and sedation to manage pain.

#### Cryptogenic organizing pneumonia

**Figure 13:**
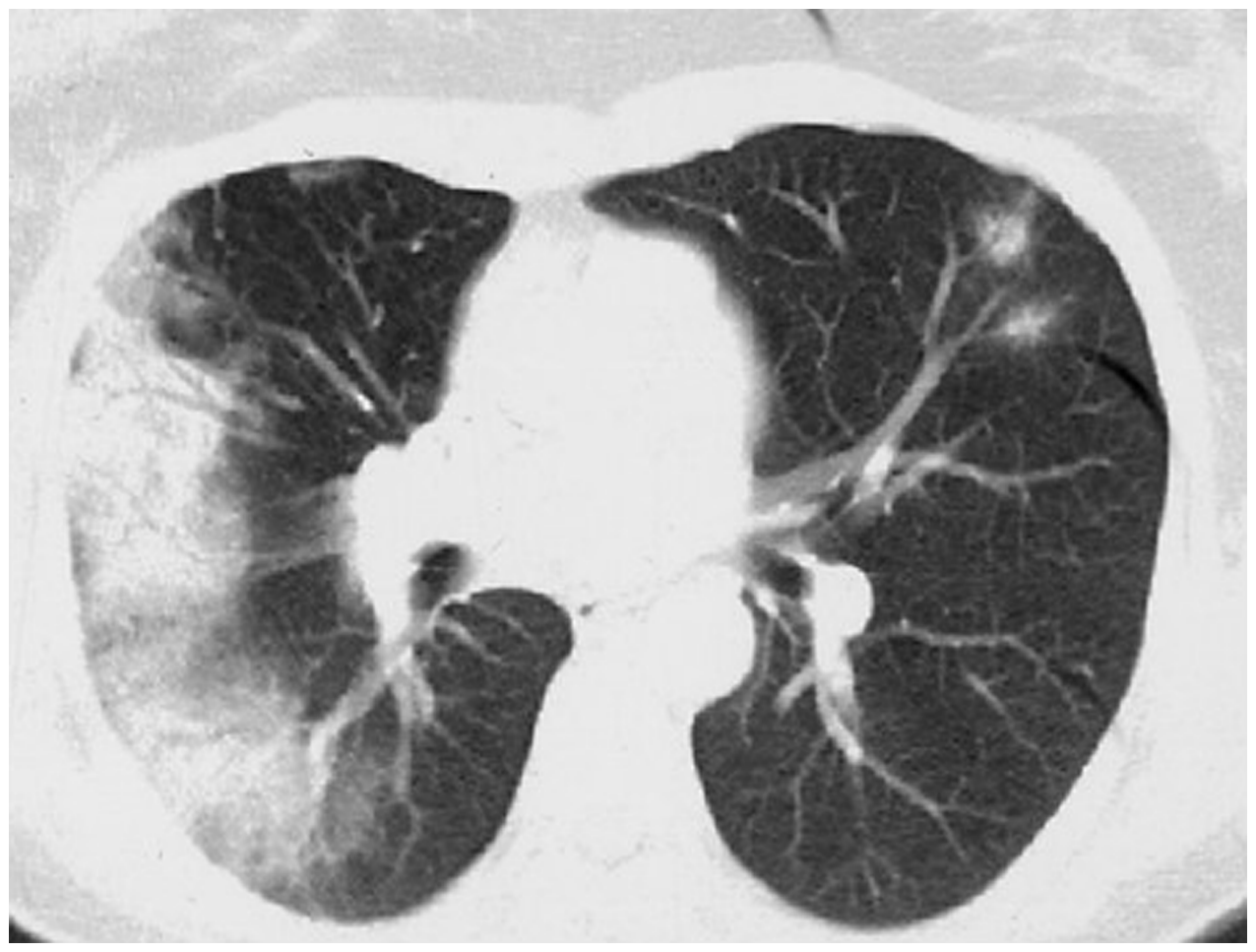
HRCT findings in cryptogenic organizing pneumonia. HRCT pattern shows areas of consolidation and ground glass with some sparing of the immediate subpleural region. This pattern is non-specific and has a wide differential including infection. Migratory (flitting) consolidation in different areas of the lung over days and weeks is a characteristic feature of COP.

**Figure 14:**
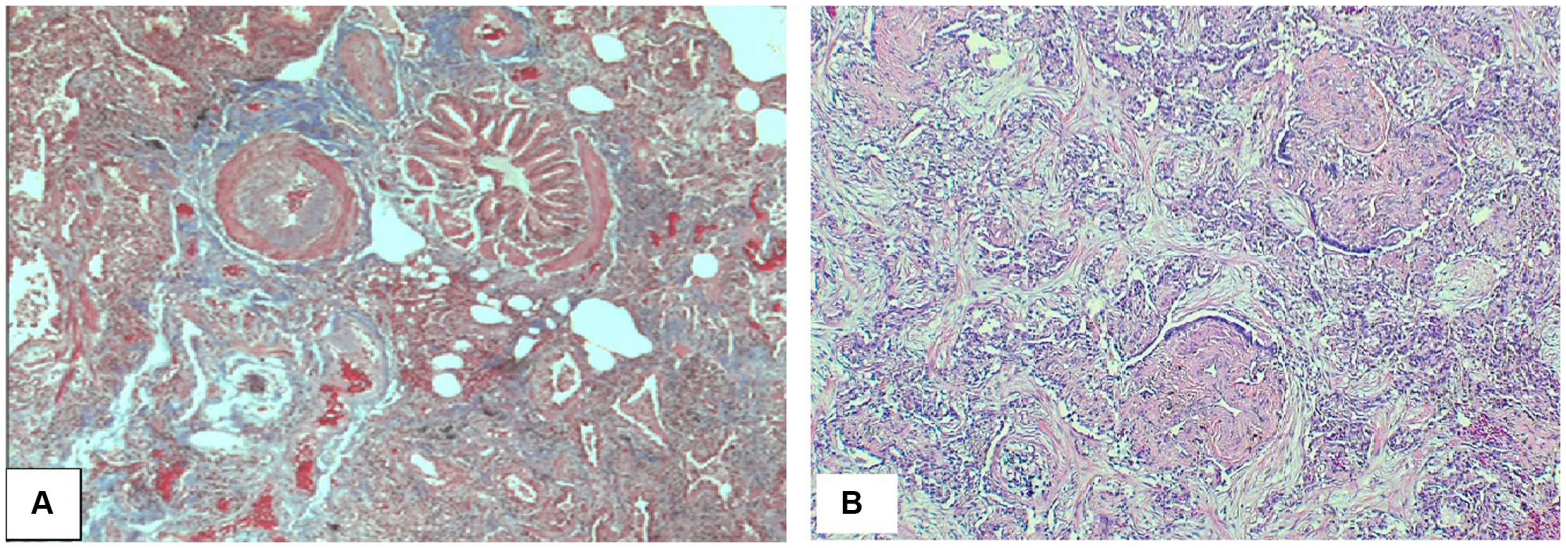
Cryptogenic organizing pneumonia. **(A)** Peribronchial connective tissue and plugs of fibroblastic connective tissue. **(B)** Interstitial reactive chronic inflammatory infiltrate with buds of myxoid granulation tissue into alveolar airspaces (so-called Masson bodies) consistent with cryptogenic organizing pneumonia. Magnification: Trichrome stain ×40 **(A)**, H&E ×125 **(B)**.

Organizing pneumonia (OP) and diffuse alveolar damage are the two main histological findings in acute lung injury. OP can represent a primary form of interstitial lung disease (ILD) and can be idiopathic in which the term cryptogenic OP (COP) is applicable, previously known as bronchiolitis obliterans organizing pneumonia (BOOP) (19, 21). OP can also be associated with connective tissue disease such as rheumatoid arthritis, systemic lupus erythematosus, scleroderma, dermatomyositis, or drugs such as amiodarone. Characteristic radiological and histological features of COP are described in Figures 13, 14.

#### Idiopathic pulmonary fibrosis and sarcoidosis

Idiopathic pulmonary fibrosis (IPF) is the most common ILD with limited response to anti-fibrotic treatment and poor prognosis. Sarcoidosis is a multiorgan granulomatous disease of unknown etiology and the lung is the most common organ affected. Mild alveolar eosinophilia may be observed in both these diseases.

## Conclusion

This overview discussed current knowledge on epidemiology, clinical manifestations, laboratory tests, histopathology, imaging, and treatment of AEP and CEP. AEP is a rare and potentially life-threatening disease with immediate response to steroid treatment and uncommon relapse. CEP frequently requires prolonged steroid treatment and may lead to persistent impairment of pulmonary function. In addition, other eosinophilic lung diseases have been described highlighting histopathological and imaging findings that can facilitate differential diagnosis and clinical assessment.

## Author contributions

RC: Conceptualization, Writing – original draft. FP: Supervision, Writing – review & editing. EM: Data curation, Writing – review & editing. AR: Data curation, Methodology, Writing – review & editing. NH: Data curation, Supervision, Writing – review & editing.
